# The Effect of Frailty on Balance, Fear of Falling, and Dual-Task Performance in Individuals with Type 2 DM

**DOI:** 10.3390/life15010025

**Published:** 2024-12-30

**Authors:** Meral Sertel, Eylem Tütün Yümin, Merve Bilgin, Hanife Büşra Hekimoğlu, Sinem Özyün, Fatma Nur Körlük

**Affiliations:** 1 Department of Physical Therapy and Rehabilitation, Faculty of Health Sciences, Bursa Uludag University, Bursa 16059, Türkiye; 2 Department of Physical Therapy and Rehabilitation, Faculty of Health Sciences, Bolu Abant İzzet Baysal University, Bolu 14030, Türkiye; tutun_e@ibu.edu.tr; 3 Department of Physical Therapy and Rehabilitation, Institute of Graduate Studies, Bolu Abant İzzet Baysal University, Bolu 14030, Türkiye; fztmervebilgin@icloud.com (M.B.); busradurmaz.bd@gmail.com (H.B.H.); sinemozyun@gmail.com (S.Ö.); fatmanurkorluk@gmail.com (F.N.K.)

**Keywords:** type 2 DM, frailty, older adult, balance, dual task, fear of falling

## Abstract

The aim of this study was to compare balance, fear of falling, and dual-task performance in frail, pre-frail, and non-frail individuals with type 2 DM. The study included 110 voluntary individuals diagnosed with type 2 DM. Individuals with type 2 DM were divided into three groups according to the FRAIL Scale: frail (*n* = 26), pre-frail (*n* = 52), and non-frail (*n* = 32). The FRAIL Scale evaluated individuals’ frailty levels, Timed Up and Go Test evaluated dual-task performance (cognitive and motor), and Tinetti Balance and Gait Scale evaluated balance and risk of falls. Considering the Fall Efficacy Scale, Tinetti Balance and Gait Scale, TUG_standard (sec)_, TUG_cognitive (sec)_, and TUG_motor (sec)_ results of frail, pre-frail, and non-frail individuals with type 2 DM, a statistically significant difference was found between the groups (*p* < 0.05). This difference originated from the frail group. Considering the increase in old age and chronic syndromes, such as DM, it brings about, it was concluded that the early evaluation of older adults for frailty and balance was essential and that individually planned intervention could improve prognosis, reduce falls that might occur due to a loss of balance and muscle strength, and enhance the quality of life in older adults.

## 1. Introduction

Diabetes mellitus (DM) is a multifactorial chronic health problem impacted by various genetic and/or environmental conditions [1]. According to the World Health Organization, it is characterized by high blood sugar levels, causing significant damage to the heart, blood vessels, eyes, kidneys, and nerves over time. Type 2 DM is the most common form. Type 2 DM is mostly observed in adults and occurs when the body becomes resistant to insulin and cannot produce insulin in sufficient amounts [2].

The epidemiology of type 2 diabetes emerges in association with aging [3]. The prevalence of type 2 DM has increased in the aging population in the last 50 years and is expected to continue to increase in the following years [1]. Advanced age is a risk factor for diabetes, falls, and disability. Micro- and macrovascular symptoms, geriatric symptoms, and frailty that occur in older adults with diabetes are among the possible complications [3].

Frailty manifests itself with the decreased ability to cope with daily or acute stress factors, which results from a decrease in physiological reserve and system function associated with advanced age [4].

Frailty leads to a loss of homeostasis, multisystem disorders, and morbidity and mortality. Frail individuals, characterized by structural and physiological changes in the brain with aging, are at risk of cognitive impairment [5].

Increased cognitive dysfunction is also observed in individuals with type 2 DM [6,7]. Furthermore, the prevalence of falls and fear of falling is increased in diabetic individuals due to various disease-related reasons [8]. Cognitive disorders, balance, and falling disorders occur with type 2 DM and frailty syndrome. This adversely affects the quality of daily life and functionality and is of great importance for public health.

Given the unique challenges faced by individuals with Diabetes Mellitus (DM), it is important to assess frailty at an early stage in this population and investigate its impact on balance, fear of falling, dual-task performance, and other health outcomes. Frailty, as a significant determinant of health outcomes in various chronic diseases, can have a profound effect on individuals’ long-term health outcomes and quality of life [9]. Considering the growing importance of frailty in the management of chronic diseases, there is a notable gap in the literature regarding the comprehensive assessment of frailty in individuals with DM. The increased number of DM individuals reaching adulthood due to advancements in surgical and medical interventions has made this gap even more critical [10].

The aim of this study is to evaluate frailty in individuals with DM and compare balance, fear of falling, and dual-task performance between those who are frail and those who are not. Additionally, this study seeks to clarify the relationship between balance, fear of falling, and dual-task performance in this unique population, emphasizing the importance of addressing frailty in the clinical management of individuals with DM. The literature review found no study on the effects of frailty on balance, fear of falling, and dual-task performance in individuals with type 2 DM. Accordingly, our study aims to contribute to the literature by comparing balance, fear of falling, and dual-task performance in frail, pre-frail, and non-frail individuals with type 2 DM.

## 2. Materials and Methods

The present research is a cross-sectional study involving individuals living in Bolu province. The study evaluated 110 individuals diagnosed with type 2 DM between February 2023 and December 2023. The program G*power (version 3.1.1) was used for post hoc power analysis. The post hoc power analysis found the statistical significance of alpha to be 5% (effect size = 0.6372). The post-power analysis was calculated as 99% for the three groups of non-frail, pre-frail, and frail individuals with DM. The inclusion criteria were determined as follows: volunteering to participate in the study, being diagnosed with type 2 DM, being independent in walking and mobilization, and having a Mini-Mental test score of 24>. The exclusion criteria were as follows: the presence of pulmonary embolism and deep vein thrombosis in the last three months, a history of cerebral aneurysm or intracranial hemorrhage, acute retinal hemorrhage or previous ophthalmic surgery, active infection, malignancy, multiple organ failure, terminal disease, a history of fracture in the lower and upper extremities in the last three months, having neurological diseases such as Multiple Sclerosis, Parkinson, Spinal Stenosis, Ataxia, and ALS, having orthopedic diseases such as osteoporosis, postoperative ankle knee hip surgery, foot pathology deformities, lower extremity contractures, having psychiatric diseases (depression, panic attacks etc.), having severe hearing and vision loss, being diagnosed with Alzheimer’s, dementia, Diabetic Neuropathy, and benign paroxysmal positional vertigo, terminal disease states, alcoholism, and those using medications that negatively affect balance and walking.

All evaluations were performed by a physiotherapist face-to-face. All evaluations were made at one time, within a convenient time period for individuals with type 2 DM who were registered at Bolu Beşkavaklar Family Health Center and volunteered to participate in the research. The Standardized Mini-Mental Test (SMMT) was applied to determine individuals’ cognitive levels. Individuals with problems in their cognitive levels were excluded from the study. First, individuals’ sociodemographic information and disease-related values were obtained from the patients themselves and the patient registration form and recorded in the Patient Evaluation Form. The patient evaluation form questioned individuals’ sociodemographic information (name-surname, address, telephone, age, sex, weight, height, body mass index, marital status, educational status, alcohol consumption, and smoking, etc.), type 2 DM-related symptoms and medical information, and disease-related values (duration of diagnosis, fasting and postprandial blood sugar and glycated hemoglobin (HbA1c) values, use of insulin, information on the medications used, presence of chronic complications due to diabetes, hospitalization due to diabetes or its complications in the last year). The FRAIL Scale was used to determine individuals’ frailty levels, the Timed Up and Go Test was used to assess dual-task performance (cognitive and motor), and the Tinetti Balance and Gait Scale was used to evaluate balance and the risk of falls.

### 2.1. Mini-Mental Test

This test is available in two different forms: a form that can be applied to literate individuals and a form that can be applied to illiterate individuals. The Standardized Mini-Mental Test (SMMT) was applied to individuals who were at least primary school graduates, and the Mini-Mental Test (MMT) was applied to illiterate individuals. The MMT was developed by Folstein et al. in 1975 as a short, useful, and standardized assessment method that can be used to determine cognitive levels and is a test that can be applied in outpatient clinics or at bedside in a period of 10 min. It consists of eleven items gathered under five main headings (orientation, registration, attention and calculation, recall, and language) and is evaluated out of a total score of 30. Güngen conducted the validity and reliability study of the test in Turkish in 2002 [11].

### 2.2. FRAIL Scale

The study used the Turkish adaptation of the FRAIL Scale [12] published by Morley et al. in 2012, which was performed by Dr. Ben Azir Begum Hymabaccus Muradi in 2017, to determine individuals’ frailty and frailty levels [13]. The FRAIL Scale has been adapted in many countries and proven to be an effective method for detecting frailty. The five-item FRAIL Scale receives 0 or 1 point depending on patients’ answers; a total score of 0 points is considered non-frail, a score of 1–2 points is considered pre-frail, and a score >2 is considered frail. This scale allows for evaluation by questioning the patient’s fatigue, resistance, mobility, weight loss, and other diseases [13].

### 2.3. Timed up and Go (TUG) Test

The TUG Test is applied to evaluate patients for balance and the risk of falls. Dual-task performance can also be assessed with additional cognitive and motor tasks added to the test. A standard chair was used for the test. First, the patient was asked to sit leaning on the chair. Then, the patient was asked to stand up, walk with regular steps a predetermined distance of 3 m, and then return at the end of 3 m and sit on the chair. The patient’s walking time during the test was recorded in seconds with a stopwatch. The average value was obtained by repeating the test three times.

**As an additional cognitive task for the Timed Up and Go Test,** individuals were asked to count backward from 50, 2 by 2, during the TUG Test. The elapsed time was recorded in seconds, and the test was repeated three times to obtain the average result.

**As an additional motor task for the Timed Up and Go Test,** individuals were asked to carry a tray with filled glasses that would not break during the TUG Test. The elapsed time was recorded in seconds, and the test was repeated three times to obtain the average result [14].

### 2.4. Tinetti Balance and Gait Scale

The Tinetti Balance and Gait Test was first developed by Mary Tinetti under the name of Performance-Oriented Assessment of Mobility Problems in Elderly Patients (POMA) to evaluate patients at a high risk of falling [15]. Afterward, it was developed and named the Tinetti Gait and Balance Assessment Tool. Ağırcan performed the validity and reliability study of the test in Turkish in 2009 [16]. It is based on the principle of evaluating patients by scoring them according to their performance during activities according to the predetermined criteria. It evaluates functions in two sections: gait and balance subscale. The evaluated activities include balance-related activities, such as sitting balance, standing up suddenly, standing, and standing with eyes closed, and gait-related activities, such as gait initiation, step length, step symmetry, deviations during walking, and trunk sway during walking. As a result of the evaluation made through observation, the scoring is as follows:

2 points: the specified movement is performed correctly.

1 point: the specified movement is performed with adaptations.

0 points: the movement is not performed.

The total maximum score is 28, with the maximum score for the balance subscale being 16 and the maximum score for the gait subscale being 12. Low scores indicate impaired balance. Scores below 19 points are related to a high risk of falling, scores between 19 and 23 points are related to a medium risk of falling, and scores of 24 points and above are related to a low risk of falling. If a score is below nineteen points, the risk of falling increases fivefold [17].

### 2.5. Fall Efficacy Scale

The Fall Efficacy Scale was developed by Tinetti et al. [18]. Ulus et al. conducted its validity and reliability study in Turkish in 2012 [19]. The scale includes 10 items questioning how confident individuals are about falling during activities of daily living. These items include taking a bath or shower, reaching for shelves, walking around the house, preparing meals that do not require carrying heavy or hot objects, getting into and out of bed, answering the door or phone, sitting in and getting up from a chair, dressing and undressing, personal care, and entering/leaving the toilet. Each item is scored between 1 (I am very confident) and 10 (I am not confident at all). It is understood that individuals receiving a high overall score have low self-confidence in falling [19].

### 2.6. Statistical Analysis

Statistical analysis was conducted using the package IBM SPSS Statistics 26.0 (SPSS Inc, Chicago, IL, USA). As descriptive statistics, frequency and percentage are given for nominal and ordinal variables, median and IQR (interquartile range) are presented for non-normally distributed numerical variables, and mean and standard deviation are presented for normally distributed numerical variables. The suitability of the variables to normal distribution was examined using visual (histogram and probability graphs) and analytical methods (Shapiro–Wilk test). When parametric test assumptions were met, intergroup comparisons were made by the “one-way ANOVA test”. “Tukey’s test” or “Tamhane’s test” was used for multiple pairwise comparisons. When parametric test assumptions were not met, differences between groups were evaluated by the “Kruskal–Wallis test” and in multiple pairwise comparisons by the Mann–Whitney U Test considering the “Bonferroni correction”. The limit of statistical significance was accepted to be *p* < 0.05. Differences between the groups in pre-treatment and post-treatment evaluations were examined using the “Paired *t*-test” and “Wilcoxon signed-rank test”.

## 3. Results

One hundred and ten individuals with type 2 DM participated in the study. Individuals were divided into three groups according to the FRAIL scale: non-frail, pre-frail, and frail. The non-frail group consisted of 32 individuals, the pre-frail group consisted of 52 individuals, and the frail group consisted of 26 individuals. The average age of the non-frail group was 64.65 ± 10.62 years, the average age of the pre-frail group was 64.61 ± 10.24 years, and the average age of the frail group was 70.00 ± 10.00 years. No significant difference was observed between the average ages of the groups (*p* > 0.05) (Table 1). Likewise, there was no statistically significant difference between the groups in height and weight parameters (*p* > 0.05) (Table 1). Table 2 lists the information of individuals with type 2 DM, including their marital status, educational status, alcohol consumption, smoking, use of oral anti-DM agents, insulin use, and falls. No statistical difference was identified between the groups in terms of falls, frequency of falls, or educational status (*p* < 0.05). Falls and frequency of falls were found to be higher in frail individuals (Table 2).

Table 3 contains the disease-related values of older adults with type 2 DM, such as DM diagnosis duration, HBA1C, and fasting and postprandial blood sugar, and there is no statistical difference in these values between the groups (*p* > 0.05).

Considering the Fall Efficacy Scale, Tinetti Balance and Gait Scale (Table 4), TUG_standard (sec)_, TUG_cognitive (sec)_, and TUG_motor (sec)_ (Table 5) results of frail, pre-frail, and non-frail individuals with type 2 DM, no statistically significant difference was found between the groups (*p* < 0.05). The difference originated from the frail group.

## 4. Discussion

Our study investigating the effects of frailty, one of the geriatric syndromes, on balance, fear of falling, and dual-task performance in individuals with type 2 DM determined that frail individuals with type 2 DM have poor balance, a high fear of falling, and low motor and cognitive dual-task performance.

Frailty and muscle loss are considered important new complications of diabetes and significant risk factors for disability [3]. Frailty is now recognized as an important risk factor in the increased death and disability risk in older adults with diabetes [20]. Numerous studies have revealed that frailty is closely associated with adverse outcomes such as hospitalization, disability, need for care, and death, which are common in patients with diabetes. Data obtained from a cross-sectional study conducted in primary care health service settings showed that individuals with diabetes (aged between 65 and 74) had poorer physical performance than their peers without diabetes [21]. A study demonstrated that frailty is an emerging high-impact complication of diabetes. The decrease in muscle mass, strength, and function associated with diabetes was shown to cause frailty and, eventually, disability. Frailty represents a dynamic process progressing from a robust to a prefrail stage, then to frailty, and finally to disability. Hence, a multimodal intervention involving adequate nutrition, exercise training, good glycemic control, and the use of appropriate hypoglycemic medications may help delay or prevent progression to disability [3].

The rapid rise of Type 2 DM and associated complications shows clinically important gender differences [22]. Frailty is more common in older women. Interestingly, regardless of age or level of frailty, older frail women have better survival compared to men [23]. A 2018 study of 3079 older adults from the 2007–2010 National Health and Nutrition Examination Survey (NHANES) database found that frailty was more common in women (8.8% women, 5.4% men). Important risk factors for frailty in women were obesity, physical inactivity, sleeping <6 h, a family history of diabetes and heart attack, and a history of hospitalization [24]. In a meta-analysis of 240 studies from 62 countries worldwide, frailty and prefrailty were more common in women [25]. A systematic review of adults over the age of 65 living in the community also found that frailty is more common in women than in men [26]. A study of community-dwelling adults using seven different frailty scales found that women had higher frailty scores than men [27]. As a result of the study, similar to the literature, it was observed that the majority of individuals in the frail and pre-frail groups were women.

### 4.1. Balance

In type 2 DM, chronic complications mostly impact the cardiovascular, musculoskeletal, and nervous systems. Neuropathies, which occur particularly on the nervous system, cause numbness, foot and skin problems, vision problems, decreased muscle strength, a loss of proprioception, antalgic gait, balance problems, and severe falling problems [28].

In their study conducted in 2022, Song et al. [29] evaluated frailty in older adults with type 2 DM using the Fried Frailty Phenotype Scale. They noted that frailty increased with advancing age and found worse body balance in the presence of frailty. Frail older adults with type 2 diabetes were also found to have a poor coordination ability.

The study conducted by Casals et al. on older adults with type 2 diabetes in 2018 found that the prevalence of frailty syndrome was higher in patients with diabetes compared to the general population over the age of 65. Frailty was shown to be associated with poorer balance and lower independence when carrying out activities of daily living [30].

In their study conducted in 2016 on community-dwelling older women with type 2 diabetes, Moreira et al. found fear of falling, frailty, and dynamic balance to be associated with functional mobility and gait disorders. The study reported a high fear of falling in this population [31].

Wang et al. researched the relationship between frailty and the risk of falls in patients with diabetes in 2020. This study revealed a significant relationship between frailty and the risk of falls in individuals with type 2 diabetes. The relationship between frailty and the risk of falls varied in balance performance in subgroups (semi-tandem standing and full tandem standing), and individuals with poor balance performance were shown to be at higher risk of falling than individuals with excellent balance performance [32].

In their study performed in 2020, Yokoyama et al. associated lower grip strength, as a factor predicting frailty, with significantly slower walking speed, poorer balance, and lower balance scores [33].

In their case-control study performed in 2022 on older adults with type 2 diabetes, Lin et al. found static/dynamic balance to be significantly poorer in the T2DM group than controls. This study found frailty to be significantly higher in patients with T2DM. It was stated that the neurological impact of T2DM impacts physical performance, including balance and walking speed, and that, therefore, these falls may accelerate the occurrence of frailty [34].

As a result of the present study, the balance evaluation with the Tinetti Balance and Gait Scale in frail, pre-frail, and non-frail individuals with type 2 DM determined lower balance scores in frail individuals with type 2 DM. The result, similar to the literature, showed that frailty may adversely affect balance performance in individuals with type 2 DM. In line with this result, we think that balance performance should be evaluated along with frailty when evaluating frail individuals with type 2 DM and that it is essential to prevent possible balance problems and falls. Diabetes mellitus may cause deterioration in postural balance due to both afferent (sensory) and efferent (motor) nervous system involvement, and consequently, postural instability may occur. Musculoskeletal disorders occur when a syndrome, such as frailty, which results from the combination of several impaired physiological mechanisms impacting multiple organs and systems, is added to this condition. We also think that the early diagnosis of frailty in older individuals with diabetes should be a routine clinical practice to facilitate interventions.

### 4.2. Fear of Falling

Older adults with type 2 diabetes have a significantly higher incidence of falls compared to those without type 2 diabetes [35]. When frailty syndrome is added to a chronic disease, such as diabetes, in older adults, it becomes a significant risk factor for muscle loss, balance, posture problems, and disability [3]. In 2020, Vongsirinavarat et al. evaluated the fear of falling, lower extremity muscle strength, and physical and balance performance in older adults with DM. The study showed that the fear of falling was higher in older adults with DM and that the fear of falling increased significantly with the increase in unsuccessful performances in the sensory interaction and balance test [36].

The study by İdiz et al. aimed to determine factors related to frailty in older adults diagnosed with type 2 DM. The study found the fear of falling score to be significantly higher in the frail group than in the non-frail group [37]. Moreira et al. conducted a study aiming to determine factors related to the fear of falling based on the data obtained from the Brazilian Elderly Frailty Study. The study found the prevalence of the fear of falling to be significantly higher in older adults with diabetes and reported that frailty was strongly related to the fear of falling in older adults with and without diabetes [38]. İdiz et al. conducted a study on the factors affecting frailty in older adults with type 2 DM. Individuals were examined in two groups: frail and non-frail. Sleep problems, fear of falling, and urinary incontinence scores were significantly higher in the frail group than in the non-frail group [37].

In a study carried out with frail older adults, Sadjapong et al. examined risk factors associated with frailty. This study showed that advanced age, low BMI, unemployment, a history of falls, DM, and malnutrition factors caused a high fear of falling [39]. Kirkwood et al. evaluated gait parameters in older women with and without DM who were classified as frail, pre-frail, and non-frail according to the Fried phenotype. The study found that the history of falling and the fear of falling were similar in all groups [40].

In another study published in 2021, Qin et al. examined the relationship between frailty and the fear of falling in community-dwelling older adults in China and observed that individuals with a fear of falling were 7.2 times more likely to be frail. The findings demonstrated that the fear of falling should be evaluated regularly in clinical practice to help identify older adults at risk of frailty [41].

A meta-analysis examined 19 studies and analyzed the prevalence of falls and frailty syndrome, as well as the relationship between them, in a population of 6724 older adults. As a result, the prevalence of falls among frail older adults was found to range between 6.7% and 44%. Furthermore, it was emphasized that there is evidence supporting the association between falls and the presence of frailty in older adults [42]. Studies have shown that frailty leads to recurrent falls in older adults, who are more likely to experience falls in pre-frailty [43].

The current study evaluated the risk of falls using the International Fall Efficacy Scale and found that frail individuals with type 2 DM had higher fall risk values, similar to studies in the literature. We think that personal and environmental risk factors should be evaluated, in addition to the routine evaluation of parameters such as sensation, balance, and muscle strength, and necessary protective measures should be taken in order to prevent the risk of falls in frail individuals with type 2 DM.

### 4.3. Dual Task

Aging is related to decreased attention capacity. Aging increases the cognitive demands of simultaneously performed tasks, causing difficulties shifting focus between tasks [44]. One of the functional effects of the age-related decrease in attention capacity is the increased risk of falls due to postural instability during dual tasking (e.g., talking while walking increases the likelihood of falls) [45]. Indeed, individuals with type 2 DM are at higher risk of falling than the healthy population. A previous study revealed that dual tasks adversely affected gait parameters in individuals with type 2 DM. The researchers suggested that impaired dual-task performance caused an increased incidence of falls in individuals with type 2 DM [46]. Additionally, the walking speed, cadence, and stepping time of frail older participants were more affected by dual tasks [47,48,49].

In a study conducted by Giusti Rossi et al. with older adults, they were divided into non-frail, pre-frail, and frail individuals, and the relationship of frailty syndrome with physical activity and dual-task performance was examined. Although the study found a worse physical activity level in the frail group, it found no difference between the groups in cognitive terms [50]. Piche et al. planned a study to evaluate the impacts of age, sex, falls, and frailty on cognitive and motor parameters in dual-task walking. This study revealed that the complex interaction between frailty, age, sex, and falls impacts dual tasking mainly through motor and cognitive performance. Parameters such as frailty*age or frailty*sex affect dual tasking [51].

Smith et al. carried out a study to examine functional life, executive functions, dual-task performance, and posture in older adults with type 2 diabetes. This study revealed that type 2 diabetes causes reduced stability when performing multiple tasks and may show deficits in executive function areas [52].

In their systematic review, Omana et al. revealed that individuals with type 1 and type 2 DM displayed poorer gait characteristics (slower walking speed, shorter step length, increased time spent on double support, wider steps, and increased variability of step length) compared to the group without diabetes both without tasks and in dual tasks. They reported that individuals with diabetes had a slower walking speed and a shorter step length [53].

Tang et al., in their study, planned to include non-frail and pre-frail elderly individuals living in the community; they found a higher incidence of falling in the pre-frail group. In addition, the TUG standard, TUG motor, and TTUG cognitive averages were found to be higher in pre-frail elderly individuals [54]. Giusti et al., in their study conducted on healthy, frail, and pre-frail elderly individuals, demonstrated that TUG performance is affected by frailty syndrome. The presence of frailty syndrome was reported to affect TUG dual-task performance [50].

When studies in the literature are reviewed, it is seen that the effects of dual tasking on parameters such as age, sex, frailty, cognitive status, balance, posture, and gait in older adults or individuals with DM have been researched. Our study evaluated the effect of dual tasks on frailty in older adults with type 2 DM. The literature review found no study conducted in this field. In this respect, we believe that the results of the present study are valuable. Our study found cognitive and motor dual-task performance to be lower in frail older adults with type 2 DM than in other groups. This suggests that dual tasking, which is the mechanism supporting balance, is more affected in frail individuals with type 2 DM, and this finding provides preliminary evidence that dual tasking disrupts balance in both frail and older adults with DM.

## 5. Conclusions

As a result, we think that it will be essential to evaluate in detail individuals, especially for frailty, balance, and dual-task performance, due to an increase in old age and the accompanying metabolic and chronic syndromes, such as DM. Thus, we believe that frailty screening, early diagnosis, and appropriate individually planned intervention can improve prognosis in older adults, reduce falls that may occur due to a loss of balance and muscle strength, and enhance the quality of life. Future research should focus on prevention strategies and rehabilitation programs for frail patients to prevent patients with Type 2 DM from becoming frail.

## 6. Limitations of the Study

The study examined the effects of frailty on balance, fall risk, and dual-task parameters in individuals with Type 2 DM. The main goal of the study was to demonstrate whether individuals with DM are more affected by frailty symptoms. Since there was no control group (asymptomatic older adults) in this study, the results were presented regarding the effects of frailty rather than the effects of DM. Therefore, it is recommended to add a control group in future studies to reveal the effects of DM.

Although the sample size of this study was found to be adequate by the power analysis, future studies should be conducted with larger sample sizes. In future studies, the effects of frailty in Type 2 DM can be investigated by using regression analyses with larger samples. Additionally, in this study, since patients with diabetic neuropathy were excluded, sensations such as plantar sensation, vibratory sensation of the lateral and medial malleolus, and deep sensation of the ankle joint were not evaluated. Sensory assessments are characteristic risk factors for falls in patients with type 2 diabetes. The limitation of this study is that sensory assessments were not performed in the study.

## Figures and Tables

**Table 1:** Individuals’ socio-demographic information.

	Non-Frail (*n* = 32)	Pre-Frail (*n* = 52)	Frail (*n* = 26)	
	Mean ± SD (Min–Max)	Mean ± SD (Min–Max)	Mean ± SD (Min–Max)	*p*
Age (years)	64.65 ± 10.62 (30.00–83.00)	64.61 ± 10.24 (48.00–91.00)	70.00 ± 10.00 (48.00–89.00)	0.072
Height (cm)	166.18 ± 7.37 (149.00–180.00)	163.05 ± 7.59 (150.00–180.00)	164.96 ± 10.43 (140.00–179.00)	0.231
Weight (kg)	77.00 ± 9.06 (59.00–95.00)	80.07 ± 13.65 (60.00–120.00)	82.07 ± 11.52 (60.00–107.00)	0.264

**Table 2:** Individuals’ clinical characteristics.

		Non-Frail (*n* = 32)	Pre-Frail (*n* = 52)	Frail (*n* = 26)	*p*
Gender	Male	21 (65.6)	19 (36.5)	9 (34.6)	0.017 *
	Female	11 (34.4)	33 (63.5)	17 (65.4)	
Marital status, *n* (%)	Married	27 (33.3)	36 (44.4)	18 (22.2)	0.469
	Single	5 (17.9)	16 (53.5)	8 (28.6)	
Educational status *n* (%)	Illiterate	1 (11.1)	2 (22.2)	6 (66.7)	0.003 *
	Primary school	13 (26.0)	28 (56.0)	9 (18.0)	
	Secondary school	2 (20.0)	3 (30.0)	5 (50.0)	
	High school	6 (27.3)	10 (45.5)	6 (27.3)	
	University	10 (52.6)	9 (47.4)	-	
Alcohol consumption *n* (%)	Yes	2 (66.7)	-	1 (33.3)	0.237
	No	28 (26.9)	51 (49.0)	25 (24.0)	
	Quit	2 (66.7)	1 (33.3)	-	
Smoking *n* (%)	Yes	6 (42.9)	6 (42.9)	2 (14.3)	0.328
	No	17 (23.6)	34 (47.2)	21 (29.2)	
	Quit	8 (34.8)	12 (52.2)	4 (14.0)	
Use of Oral Anti-DM Agents *n* (%)	Yes	26 (28.9)	43 (47.8)	21 (23.3)	0.974
	No	6 (30.0)	9 (45.0)	5 (25.0)	
Insulin Use *n* (%)	Yes	9 (32.1)	10 (35.7)	9 (32.1)	0.312
	No	23 (28.0)	42 (51.2)	17 (20.7)	
Falls	Yes	1 (3.8)	16 (61.5)	9 (34.6)	0.005 *
	No	31 (36.9)	36 (42.9)	17 (20.2)	
Frequency of Falls	Never	31 (35.2)	39 (44.3)	18 (20.5)	0.016 *
	3–4 Times	-	13 (65.0)	7 (35.0)	
	4–6 Times	-	-	1 (100)	
	More than 6 times	-	-	1 (100)	
Fear of Falling	There is	6 (17.1)	19 (54.3)	10 (28.6)	0.147
	There is not	22 (35.5)	28 (45.2)	12 (19.4)	

* *p* < 0.05.

**Table 3:** Disease-related information, DM-related information of individuals.

	Non-Frail (*n* = 32)	Pre-Frail (*n* = 52)	Frail (*n* = 26)	
	Mean ± SD (Min–Max)	Mean ± SD (Min–Max)	Mean ± SD (Min–Max)	*p*
DM diagnosis duration	8.70 ± 5.92 (0.06–20.00)	10.43 ± 8.96 (0.02–43.00)	9.92 ±7.36 (1.00–25.00)	0.624
HBA1C	6.24 ± 1.02 (4.70–7.50)	6.52 ± 0.77 (5.50–7.70)	7.52 ± 0.90 (7.00–8.56)	0.164
Fasting Blood Sugar	127.00 ± 21.33 (81.00–157.00)	143.44 ± 67.71 (53.00–325.00)	184.00 ± 77.05 (110.00–320.00)	0.134
Postprandial Blood Sugar	169.70 ± 42.48 (100.00–240.00)	164.00 ± 52.42 (85.00–283)	211.14 ± 86.90 (72.00–310.00)	0.202

**Table 4:** Intergroup comparison of individuals’ fall and balance results.

	Non-Frail (*n* = 32)			Pre-Frail (*n* = 52)			Frail (*n* = 26)			F	*p*
	Mean ± SD (Min–Max)			Mean ± SD (Min–Max)			Mean ± SD (Min–Max)				
Mini-Mental Test	25.40 ± 3.61 (17.00–30.00)			25.88 ± 3.29 (17.00–30.00)			24.50 ± 4.16 (15.00–30.00)			1.274	0.284
Fall Efficacy Scale	14.22 ± 8.55 (10.00–38.00)	25.88 ± 3.29 (17.00–30.00)	24.50 ± 4.16 (15.00–30.00)	14.78 ± 7.80 (10.00–40.00)	25.88 ± 3.29 (17.00–30.00)	24.50 ± 4.16 (15.00–30.00)	22.63 ± 14.05 (10.00–56.00) *	25.88 ± 3.29 (17.00–30.00)	24.50 ± 4.16 (15.00–30.00)	5.809	0.004
Tinetti overall	33.48 ± 3.34 (20.00–35.00)			32.32 ± 4.70 (9.00–35.00)			25.61 ± 8.02 (8.00–35.00) *			17.240	0.000
Tinetti balance	24.82 ± 2.82 (14.00–26.00)			23.83 ± 4.03 (7.00–29.00)			19.00 ± 5.87 (5.00–26.00) *			14.744	0.000
Tinetti gait	8.65 ± 0.93 (6.00–9.00)			8.91 ± 3.77 (2.00–33.00)			6.061 ± 2.63 (1.00–9.00) *			5.499	0.005

* *p* < 0.05; one-way ANOVA test.

**Table 5:** Intergroup comparison of individuals’ TUG results.

	Non-Frail (*n* = 32)	Pre-Frail (*n* = 52)	Frail (*n* = 26)	F	*p*
	Mean ± SD (Min–Max)	Mean ± SD (Min–Max)	Mean ± SD (Min–Max)		
TUG_standard (sec)_	11.45 ± 4.37 (7.73–31.60)	11.01 ± 2.12 (6.65–16.04)	17.09 ± 8.74 (8.16–45.60) *	13.631	0.000
TUG_cognitive (sec)_	13.86 ± 5.06 (7.74–34.80)	14.18 ± 4.30 (6.89–26.02)	22.74 ± 11.91 (9.46–51.80) *	14.209	0.000
TUG_motor (sec)_	11.88 ± 4.91 (7.18–35.00)	11.38 ± 2.69 (6.88–20.08)	18.87 ± 9.42 (7.82–42.60) *	16.286	0.000

* *p* < 0.05; one-way ANOVA test.

## Data Availability

Data will be made available on request. The data that has been used is confidential.
